# A genome-scale metabolic reconstruction of *Lysinibacillus sphaericus* unveils unexploited biotechnological potentials

**DOI:** 10.1371/journal.pone.0179666

**Published:** 2017-06-12

**Authors:** Camilo Gómez-Garzón, Alejandra Hernández-Santana, Jenny Dussán

**Affiliations:** Centro de investigaciones microbiológicas (CIMIC), Universidad de los Andes, Bogotá, Colombia; Illinois Institute of Technology, UNITED STATES

## Abstract

The toxic lineage (TL) of *Lysinibacillus sphaericus* has been extensively studied because of its potential biotechnological applications in biocontrol of mosquitoes and bioremediation of toxic metals. We previously proposed that *L*. *sphaericus* TL should be considered as a novel species based on a comparative genomic analysis. In the current work, we constructed the first manually curated metabolic reconstruction for this species on the basis of the available genomes. We elucidated the central metabolism of the proposed species and, beyond confirming the reported experimental evidence with genomic a support, we found insights to propose novel applications and traits to be considered in further studies. The strains belonging to this lineage exhibit a broad repertory of genes encoding insecticidal factors, some of them remain uncharacterized. These strains exhibit other unexploited biotechnological important traits, such as lactonases (quorum quenching), toxic metal resistance, and potential for aromatic compound degradation. In summary, this study provides a guideline for further research aimed to implement this organism in biocontrol and bioremediation. Similarly, we highlighted the unanswered questions to be responded in order to gain a deeper understanding of the *L*. *sphaericus* TL biology.

## Introduction

Since the first description of a highly entomopathogenic strain of the spore-forming bacterium *Lysinibacillus sphaericus* (as *Bacillus sphaericus* in 1978) [1], there have been an increasing interest on the potential uses of this bacterium in biocontrol. In brief, some strains of *L*. *sphaericus* exhibit high toxicity against larvae of *Aedes aegypti* and *Culex quinquefasciatus*, which are vectors of tropical diseases [2]. In addition, *L*. *sphaericus* also has been studied for its resistance to toxic metals [3,4] and for its capability to produce biosurfactants [5].

The toxicity of *L*. *sphaericus* is mainly related to the binary toxin and the mosquitocidal toxins. The binary toxin is encoded by the *binA* and *binB* genes and produced as a paracrystalline structure attached to the spore. Separately, the mosquitocidal toxins (Mtx1, 2, and 3) are synthesized by the vegetative cell [2]. However, these toxins are not produced by all *L*. *sphaericus* strains and are indeed restricted to a monophyletic group denominated toxic lineage (TL). Recently, we proposed that the TL should be considered a separated species as well as demonstrated the lack of a clear circumscription for the non-toxic strains [6].

Currently, there are sixteen *L*. *sphaericus* genomes published in the NCBI Genome database, of which a total of six strains are considered highly toxic based on numerous studies on their toxicity, physiology, metabolism, and metal resistance. [3,7]. Despite this availability of data, there are neither curated metabolic models nor revised gene annotations of this species. As described in the scientific literature, this kind of approach permits new insights into the metabolic capabilities and evolutionary history of an organism, providing a framework to interpret experimental data gathered at cellular or molecular scale [8]. This would be especially useful in the case of an organism as *L*. *sphaericus*, because it would represent a guideline to conduct studies aimed to formulate new uses; to assess its performance in environmental applications; and to conduct assays to characterize traits that may remain undescribed.

The growing amount of genomic data, along with the efficient bioinformatics methods currently available, have shifted the descriptions of microorganisms from biochemical characterization to functional genomics. [9]. The desire of understanding the relationship between the genome and the physiology of an organism has prompted the construction of metabolic models. An initial metabolic reconstruction is easily obtained from genome sequence through automatized processes. However, such a reconstruction requires the correction of many imperfections that involves the combined analysis of the available information on protein sequence, phylogeny, gene-context, and co-occurrence [10].

In order to overcome this issue, several research teams have focused their efforts on creating well-curated databases. These databases are usually organism specific and are constructed for those microorganisms with remarkable characteristics, such as pathogens, probiotics, and extremophiles [8]. Consequently, the number of databases is still too small compared to the number of organisms whose metabolic features are being studied. This is the case of the entomopathogenic strains of *L*. *sphaericus*, for which there are no curated or specific annotation databases.

In the light of this, we present a metabolic reconstruction of the *L*. *sphaericus* TL. This reconstruction was generated from a manually curated annotation and supported by previously reported experimental evidence. It highlights the traits that make this species a potential agent for biocontrol and bioremediation.

## Results

### There are tiny differences in gene content among highly toxic strains

*L*. *sphaericus* TL is the only set of strains within this group with a clear taxonomic circumscription as species [6]. The strains belonging to this group share some insecticidal factors such as the binary toxin but, on the other hand, present differences in their toxicity level [6]. These differences are probably accounted for by the presence of other toxin-encoding genes such as *mtx* and *cry* [11]. We delimited the current study to the 6 strains cataloged as highly toxic (Table 1) because they are all considered potential biocontrol agents against mosquito larvae, and constitute a nearly clonal clade with largely syntenic genomes [6]. Further references to *L*. *sphaericus* TL in this document refers to these six highly toxic strains.

**Table 1:** *L*. *sphaericus* TL. The six TL strains available in NCBI are reported here; values shown are from the NCBI Genome database.

Strain	Genome Size (bp)	GC content (%)	Total Genes	Coding Genes	RNA Genes	Pseudogenes	S-layer related genes	Accession number
**2362**	4.692.801	37.30	4,538	4,295	149	94	13	CP015224
**OT4b.25**	4.665.575	37.14	4,714	4,452	149	113	13	CP014643
**III(3)7**	4.663.526	37.16	4,721	4,485	149	87	13	CP014856
**C3-41**	4.639.821	37.15	4,886	4,772	117	85	16	CP000817
**OT4b.49**	4.668.842	37.30	4,487	4,241	107	97	15	LWHI01000000
**CBAM5**	5.152.958	37.20	5,326	4,995	242	83	12	AYKQ00000000

In the first place, we aimed to find the differences among the gene content of the highly toxic strains and to assess whether these differences could compromise essential metabolic traits or those related to their potential for biocontrol and bioremediation. We contrasted the annotations obtained by Prokka [12] with the ones obtained by the NCBI annotation pipeline [13]. The results (Table 1) showed differences in the gene content among the strains. However, the majority of these differences are related to hypothetical proteins, and none of them involves any gene evaluated in our metabolic reconstruction. Non-functional genes were associated with two cases: they were present in all the strains or, otherwise, only in one strain but together with a functional copy in the genome.

**Fig 1:**
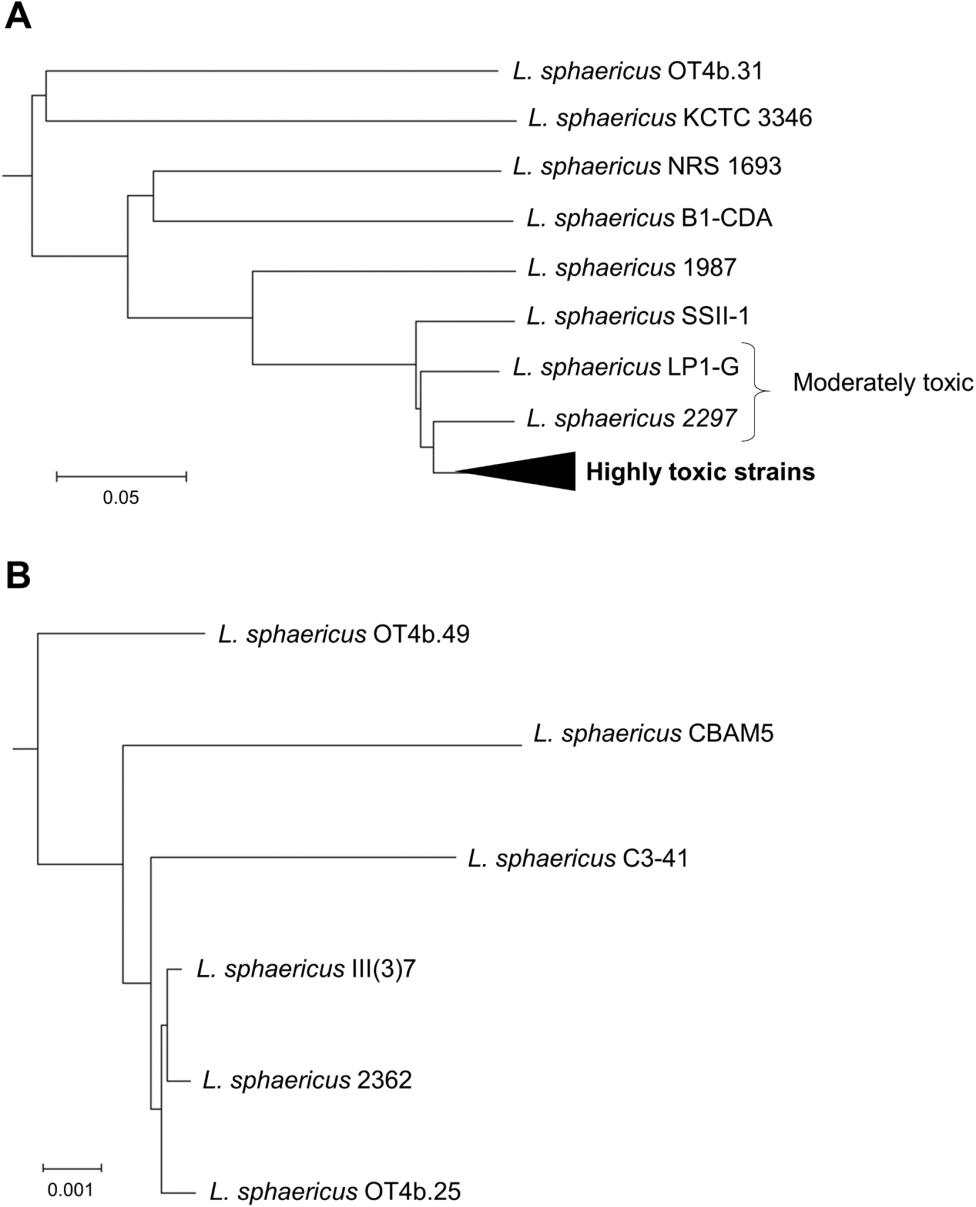
Alignment-free phylogeny reconstruction of *L*. *sphaericus*. (A) This phylogeny was constructed based on the frequency of >6-string predicted peptides from whole genome sequences. (B) The clade of highly toxic strains is shown as zoomed-in and illustrates the low diversity within this group. The outgroup (*Solibacillus silvestris* StLB046, AN AP012157) is not shown for a better image scaling. The distance is an output calculated by the CVTree 3.0 algorithm.

The visualization of those scarce differences is difficult with traditional tools such as phylogenetic trees based on alignments or clustering of orthologous genes. For instance, by the core-genome alignment of the highly toxic strains genomes, or by the alignment of commonly used single-copy markers, is not possible to obtain a tree with enough resolution to show each strain as a single branch or nodes with considerable bootstrap support [6]. Therefore, we used an alignment-free tool to construct a phylogenetic tree based on whole genome sequences [14]. The obtained tree (Fig 1) allows to observe the distances among the genomes within the toxic lineage. The topology of this phylogenetic maintains the evolutionary history as it is calculated based on the frequency of predicted oligopeptides of a fixed length [15].

Taking into account these results, we used the genome of strain 2362 as reference to conduct the annotation and to build the metabolic reconstruction. This strain is the reference *L*. *sphaericus* strain of the World Health Organization for entomopathogenic activity. Its genome was recently sequenced using PacBio technology and assembled as a single circular contig with no evidence of plasmids [16].

### Metabolic reconstruction of *L*. *sphaericus* TL

**Fig 2:**
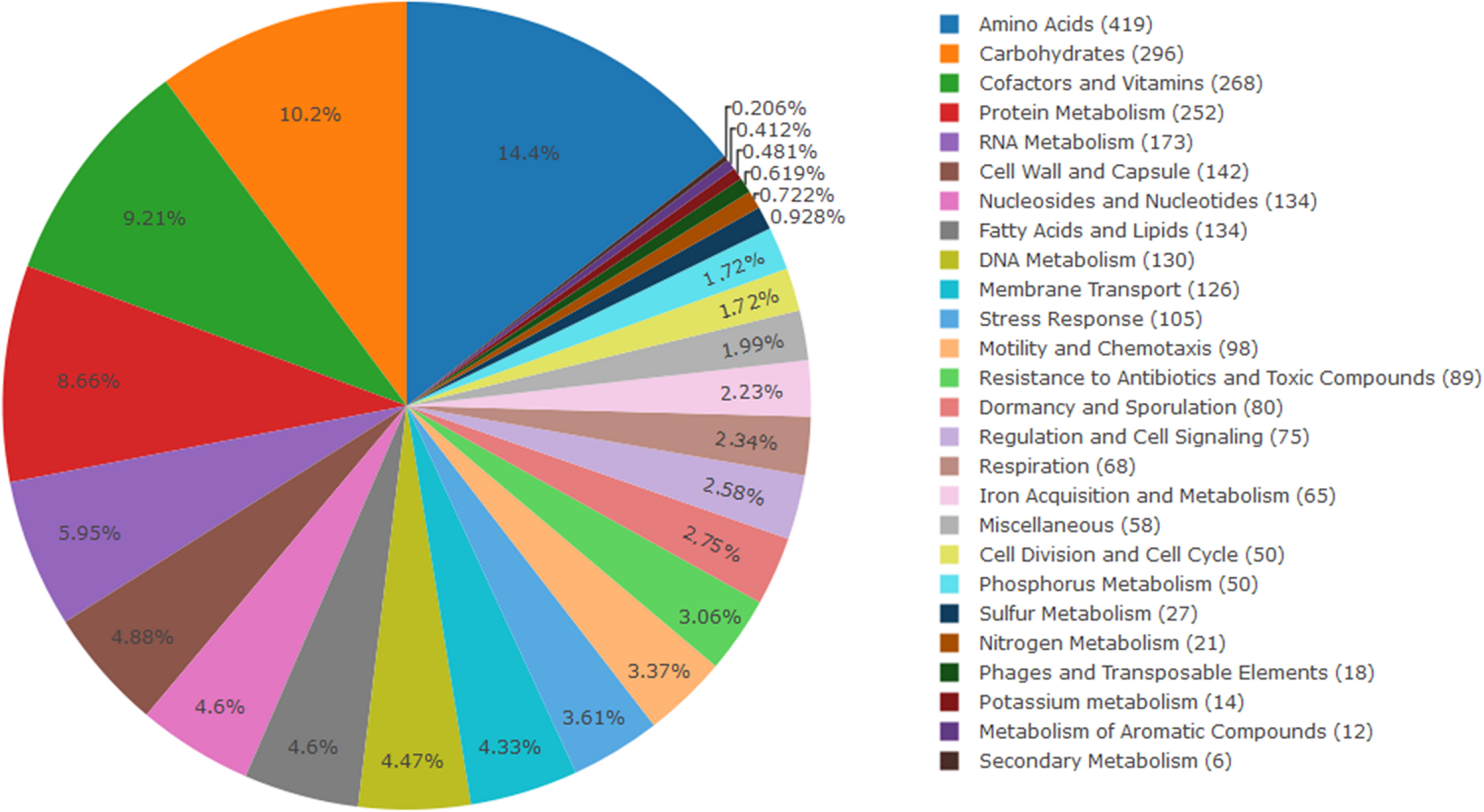
Gene content analysis of *L*. *sphaericus* TL associated to subsystems according to functional categories. The genes distribution was calculated by RAST and illustrated using the plotly package in the R statistical software.

We built a compendium of metabolic pathways of the mosquitocidal *L*. *sphaericus* 2362 by integrating the annotations obtained from the pipelines mentioned previously together with those from RAST [17,18] and KAAS [19]. In total, 2,033 genes (44%) were assigned into 456 subsystems by RAST (Fig 2) Likewise, 1,782 genes were matched to a KEGG Orthology entry by KAAS. The genes associated with the pathways herein described, such as entomopathogenicity, quorum quenching, toxic metals resistance, and the metabolism of amino acids, nitrogen and iron; were identified and their annotations, manually curated (S1 Table).

**Fig 3:**
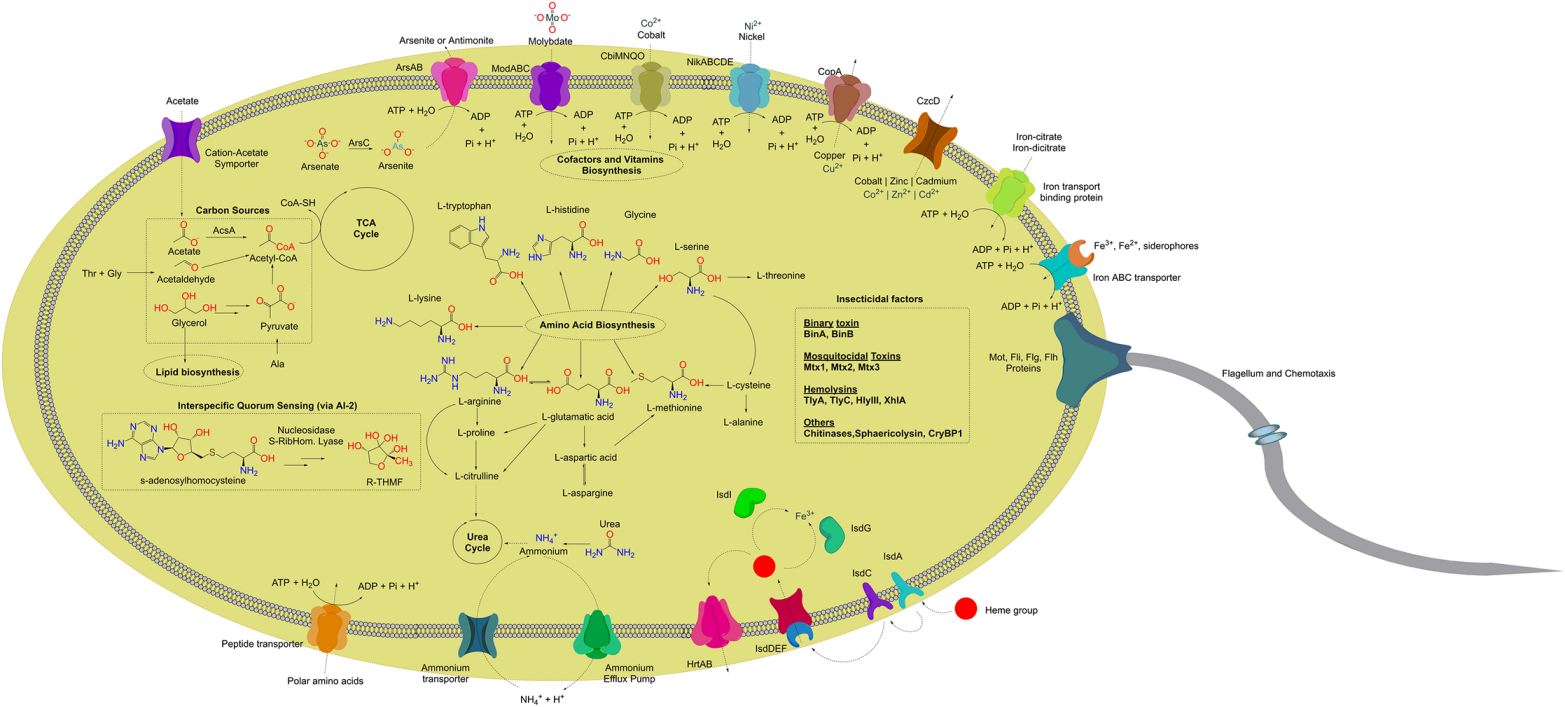
Metabolic overview representation of *L*. *sphaericus* TL. Selected mechanisms for metals transport, carbon sources metabolism, motility, and biosynthesis of amino acids and AI-2 are illustrated. All the genes involved in the depicted mechanisms are listed in S1 Table.

Subsequently, we constructed a metabolic model using the Pathologic software as part of the Pathway Tools 20.0 package [20]. In summary, 227 pathways and 1,538 reactions were assigned. In this model, 996 genes were unambiguously assigned to a reaction on the basis of matching Enzyme Code (EC) numbers, GO terms and/or names. On the other hand, 132 assignments remained as ambiguous. The putative metabolic pathways herein described are illustrated in Fig 3.

### Central metabolism: Carbon and nitrogen sources requirements

A well-known metabolic trait of *L*. *sphaericus* is its incapability to metabolize carbohydrates [6]. The culture media for this bacterium usually include acetate or glycerol instead of glucose as carbon source. This trait is conserved by all *L*. *sphaericus* groups regardless of their toxicity. According to our metabolic reconstruction, *L*. *sphaericus* is unable to metabolize carbohydrates due to lack of an enzyme (EC 5.3.1.9) that catalyzes the glucose-6-phosphate isomerisation. Acetate and glycerol are used as carbon sources and converted to acetyl-CoA, which enters the TCA cycle. Another potential carbon source might be N-acetylglucosamine, which has been proposed previously [21]. Moreover, nitrogenated compounds such as ethanolamine, threonine, and glycine may be used as alternative carbon and nitrogen sources.

The UreABCDEFG operon is present in the *L*. *sphaericus* TL genome together with genes involved in the urea cycle. This versatile set of nitrogen metabolism pathways provide *L*. *sphaericus* TL with a fitness increment under different environmental condition [21]. In addition to the genes coding for urea metabolism, other nitrogen-related genes such as *nifU*, *nosL*, nitroreductases, and regulators are present in the *L*. *sphaericus* TL genome (S1 Table). Although the annotation of some of these genes is univocal, we did not find other genes to complete operons or metabolic pathways (e.g. nitrogen fixation or nitrogenases maturation).

The amino acids that *L*. *sphaericus* TL is able to synthesize are shown in Fig 3. This bacterium requires at minimum branched amino acids (L-leucine, L-isoleucine, and L-valine), L-phenylalanine and L-tyrosine as essential nutrients. *L*. *sphaericus* TL is unable to synthesize these five amino acids due to the absence of enzymes involved in the reactions; EC 2.6.1.42 (branched chain amino acid transaminase), 3.5.1.5 (tyrosine transaminase), and 2.6.1.57 (aromatic amino acid aminotransferase).

### *L*. *sphaericus* TL is a reservoir of genes encoding insecticidal factors

The 6 highly toxic strains of *L*. *sphaericus* share 5 representative genes that encode insecticidal factors, the binary toxin (*binA*, *binB*) and the mosquitocidal toxins (*mtx1*, *mtx2*, *mtx3*). Lower toxicity strains exhibit only some of these genes and do not belong to the same clonal lineage as shown in Fig 1. The strain LP1-G is the only one that exhibits the toxins Cry48Aa and Cry49Aa but not the Mtx toxins and thus, it does not belong to the TL herein evaluated.

As previously reported, the genes coding for binary and mosquitocidal toxins are completely conserved throughout the TL group and are always embedded into genomic islands [6]. Furthermore, *L*. *sphaericus* TL has other genes that presumably could be associated with its entomotoxicity (Table 2). We found a gene that encodes a 176 aa peptide annotated by the NCBI pipeline as an ABC Transporter Permease. However, the presence of the CryBP1 (2.90e-71) as its only domain suggests a possible role in entomotoxicity. This peptide shows 60% identity with the CryBP1 toxin from *Brevibacillus laterosporus* GI-9 (AN CCF17093) and was annotated by Prokka as an insecticidal toxin.

**Table 2:** Insecticidal factor-coding genes found in *L*. *sphaericus* TL. The homologous RefSeq entry per predicted protein is the same for all toxic strains. Asterisks (*) indicate discrepancies between the proposed and NCBI annotations.

Category	Annotation	RefSeq Sequence
Toxins	Binary toxin subunit A (BinA)	WP_012291791
	Binary toxin subunit B (BinB)	WP_012291792
	Mosquitocidal toxin Mtx1	WP_036160771
	Mosquitocidal toxin Mtx2	WP_036161900
	Mosquitocidal toxin Mtx3	WP_012294440
	CryBP1 Protein	WP_012291793 *
	Sphaericolysin / Alveolysin	WP_031416422
Hemolysins	Hemolysin XhlA	WP_051563194
	Hemolysin A (TlyA)	WP_012295056 *
	Hemolysin C (TlyC)	WP_036162133 *
Chitin Metabolism	Chitin binding protein	WP_036162780
	Chitin deacetylase	WP_031419289
	Chitin deacetylase	WP_012292324
	Beta-hexosaminidase	WP_012294072

We also identified a set of hemolysin-encoding genes, which includes hemolysin A, C, D and *xhlA*. These hemolysins can lyse insect immune cells and thus, they are considered factors that contribute to the insecticidal activity [22]. The sphaericolysin-encoding gene is present in the *L*. *sphaericus* TL genomes as well. This cytolysin, homologous to the alveolysin from *Bacillus alvei* and cereolysin O from *B*. *cereus*, is an enzyme with demonstrated insecticidal activity against the German cockroach *Blattela germanica* [23]. Separately, we did not find enough genomic or experimental evidence to support necrotrophism in *L*. *sphaericus* TL (lipolytic and proteolytic activity, data not shown) as reported for the mosquitocidal *B*. *thuringiensis* [24].

**Fig 4:**
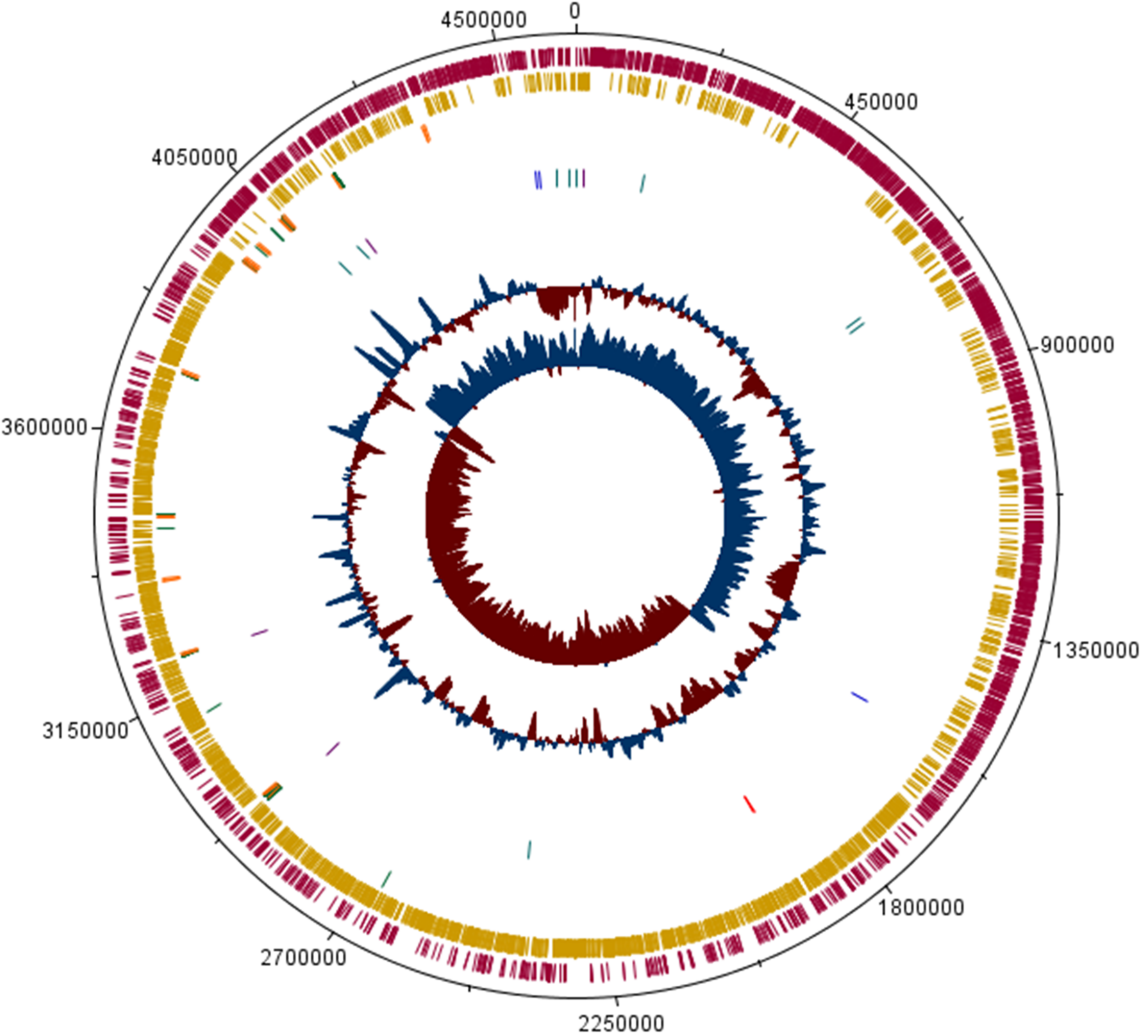
Circular plot of *L*. *sphaericus* 2362 genome generated by DNA plotter. Circles indicate, from inside outwards: GC skew; GC content; characteristic toxin-coding genes (mosquitocidal toxins in blue and binary toxin in red) and S-layer protein-coding genes on forward (dark green) and reverse strand (purple); tRNA coding genes on forward (orange) and reverse strand (green); protein-coding genes on reverse strand (ocher); and protein-coding genes on forward strand (magenta).

It is important to highlight that the S-layer protein has a presumptive role in entomotoxicity by the vegetative cells of *L*. *sphaericus* TL [25]. There are several hypotheses about how this protein works and how it is regulated. We reviewed these hypotheses in the discussion section. The number of S-layer related genes in each toxic strain is shown in Table 1 and depicted in Fig 4 along with the mosquitocidal toxins that have been experimentally validated in other studies.

### *L*. *sphaericus* TL strains exhibit resistance to several toxic metals

Besides the mosquitocidal activity, *L*. *sphaericus* has been extensively studied due to its toxic metal resistance [3]. For example, the potential for arsenic remediation of the strain B1-CDA (non-toxic) was recently shown [26]. Later, the genome sequencing of this strain confirmed this observation and revealed a broad repertory of putative mechanisms involved in toxic metal resistance [27]. However, this activity is not limited to non-toxic strains and, indeed, the dual activity of the toxic strains has been demonstrated [3,4].

**Fig 5:**
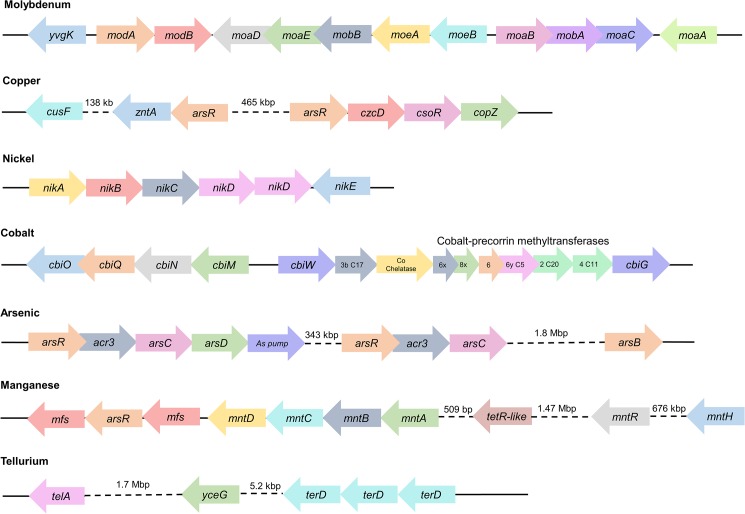
Regulons for toxic metal resistance and metabolism found in *L*. *sphaericus* TL. The gene annotations are the same as those reported for the marine bacterium *Bacillus sp*. B-14905 in the RegPrecise database. Arrows indicate the relative directions of transcription. Arrow lengths are not drawn to scale. Other metal resistance (Al, Cr, Cd) are detailed in S1 Table.

We found enough genomic evidence to support the resistance by toxic strains of *L*. *sphaericus* against arsenic, copper, tellurium, and molybdenum as well as we found genes involved in cobalt and nickel metabolism for cofactors and vitamin biosynthesis. These genes are arranged in operons (Fig 5) orthologous to already characterized operons from the marine bacterium *Bacillus sp*. B-14905 [21]. These operons encode ATP-dependent efflux pumps known as ECF transporters and, for copper and arsenic, seem to be regulated by ArsR-like transcriptional factors. The annotation of these regulons has been manually curated for *Bacillus sp*. B-14905 and is available on the RegPrecise database [28].

In addition, we found several genes encoding putative metal resistance proteins and ArsR-like and MerR-like transcriptional factors, and none of these genes exhibited a clear association with any regulons. The origin or function of those genes is still unknown as they could be the result of recombination events or remnants from evolutionary changes. Similar to this case, some genes that have no univocal annotation could also be involved in metal resistance. For instance, the gene that encodes the WP_031419065 protein (annotated by the NCBI pipeline as “hypothetical protein”) exhibits the YnbB domain, for aluminum resistance, with an e-value of 0.00.

It has been proven that S-layer proteins also contribute to metal resistance by adsorbing metal cations on the cell surface [29]. This is probably a non-specific mechanism used by *L*. *sphaericus* TL to overcome the environmental stress caused by toxic metals.

### *L*. *sphaericus* TL would be able to disrupt communication among Gram negative bacteria

To gain insights into the interactions between *L*. *sphaericus* TL and other bacteria, we explored its genome looking for quorum quenching and quorum sensing genes. We found two genes encoding the proteins with the RefSeq entries WP_036162813 and WP_031418585. In *L*. *sphaericus*, these genes are exclusive of the TL subgroup and are currently annotated as zinc dependent beta-metallo-hydrolases, a family that comprises some lactamases and acyl-homoserine lactonases (quorum quenching enzymes) [30]. The best manually reviewed hits obtained by Blast for these genes (against UniProt database) were respectively: N-acyl homoserine lactonase AiiB from *Agrobacterium radiobacter* (AN B9JPK6, 98% coverage, 42% identity, and e-value 5.8 e-72) and putative quorum-quenching lactonase YtnP from *B*. *subtilis* (AN O34760, 98% coverage, 60% identity, and e-value 4 e-113). We confirmed the presence of the locus BSPH_RS15750 (formerly Bsph_3377) in all the studied genomes. The relation between this locus and quorum quenching activity has been reported in previous studies [30]. Moreover, *L*. *sphaericus* TL exhibits the genes involved in the AI-2 biosynthesis pathway. AI-2 (autoinducer 2) is a signal molecule used in interspecific bacterial interactions, even between Gram negative and Gram positive bacteria [31].

### Motility, chemotaxis and resistance to antimicrobial agents

*L*. *sphaericus* TL strains have been reported as motile and chemotactic bacteria [32]. This evidence is supported by the genomic data since all the genes required for flagellum assembly in Gram positive bacteria are present. In contrast, motility is not a trait present in all non-toxic strains. Besides, *L*. *sphaericus* TL genomes contain genes involved in chemotactic response, such as the operon Che and methyl accepting chemotaxis proteins.

With respect to antimicrobials resistance, *L*. *sphaericus* TL showed exhibits against erythromycin from a 5 μg^.^mL^-1^ concentration in lab experiments (S2 Table). This finding can be explained by the erythromycin resistance protein (WP_012295993.1). Likewise, we found genes coding for beta-lactam resistance and a bacitracin transporter (S1 Table), but no experimental data to support this. According to our lab experience, *L*. *sphaericus* TL is susceptible to ampicillin, trimethoprim, chloramphenicol, kanamycin, rifampicin, tetracycline, and gentamycin (S2 Table).

### *Lysinibicallus sphaericus* TL has a vast array of genes encoding for iron sequesters

Iron is an essential nutrient for life. It is needed to catalyze diverse indispensable enzymatic reactions. Several genes related to iron metabolism were found in *L*. *sphaericus* TL genomes (S1 Table), mainly genes responsible for iron uptake and transport, siderophores biosynthesis, iron capture from heme and hemin groups, and iron storage.

The gene encoding for the ferric uptake regulation protein (Fur) was found. Additionally, nine possible Fur-binding boxes were identified in *L*. *sphaericus* TL genomes by searching for occurrences of the Fur high-affinity binding site defined for *Bacillus subtilis*: TGATAATNATTATCA [33]. In the same way that Harvie et al. identified Fur binding sites in the *Bacillus cereus* genome [34], we only accepted hits that were within 250 pb of an identified ORF and contained a maximum of two mismatches from the consensus sequence. The identified Fur binding regions, as well as the genes predicted to be Fur-regulated, are shown in Table 3. Fur binding boxes were found upstream of genes mainly related to iron uptake, though some genes involved in oxidative stress, such as the ones encoding for Ferredoxin NADP-reductase and thioredoxin reductase, also seem to be Fur-regulated.

**Table 3:** Genes identified in the *L*. *sphaericus* TL genomes as having upstream Fur box motifs. The identified Fur box sequences and their position in the *L*. *sphaericus* 2362 genome are shown, along with the locus tags of the genes that are no more than 250 pb downstream to the Fur box. Gene function and relative position (in bp) for each Fur box upstream are given.

Fur box sequence	Position	Locus tag	Function	Position to Fur box (bp)
tttagTGATAATGATTATCAttttt	1030906–1030920	A2J09_RS04875	iron-dicitrate ABC transporter ATP-binding	58
aaaaaTGATAATGATTATCActaaa	1030920–1030906	A2J09_RS04870	Ferredoxin-NADP reductase 3	90
gaggaTGATAATCATTATCAtcaca	1038754–1038768	A2J09_RS04910	iron ABC transporter substrate-binding protein	52
gtgagTGATAATCATTATCAattaa	1584015–1584001	A2J09_RS07705	siderophore biosynthesis protein	42
acgtaTGATAATGATTATCAattac	4038130–4038116	A2J09_RS19865	ferrichrome ABC transporter substrate-binding protein^a^	18
ttactTGATAATCATTATCAttatc	4142246–4142260	A2J09_RS20375	thioredoxin reductase	151
gataaTGATAATGATTATCAagtaa	4142260–4142246	A2J09_RS20370	alkaline phosphatase-like protein	122
gaaatTGATAATCATTATCAatata	4354641–4354655	A2J09_RS21310	Hypothetical protein^b^	42
ttaatTGATAATCATTATCGttatt	422084–422097	A2J09_RS01965	iron ABC transporter substrate-binding protein	127

^a^ Pseudogene with internal stop

^b^ Hypothetical protein with domains of an ABC-type Fe^3+^-hydroxamate transport system

Siderophores are selective Fe^3+^-chelators with an extremely high affinity for this trivalent metal ion. A putative homologue of the siderophore synthesis operon *asb* in *Bacillus anthracis* was found in the genomes of *L*. *sphaericus* TL. The operon comprises two siderophore biosynthesis proteins, a Co-A ligase, an acyl carrier protein, and a hypothetical protein associated with anthrachelin biosynthesis. Several siderophores transporter ATP-binding proteins, iron ABC transporter binding proteins, and iron ABC transporter permeases were also found in *L*. *sphaericus* TL genomes (S1 Table).

Citrate is not a high-affinity iron chelator, but it can serve as an iron-delivery agent for several bacteria [35]. Genes encoding iron-citrate and iron-dicitrate ATP binding proteins were found in the genomes of *L*. *sphaericus* TL.

Genome sequence analysis revealed four ORFs (*feuA*, *feuB*, *feuC*, and *feuD*) organized as an operon encoding a periplasmic solute-binding protein (FeuA), permeases (FeuB and FeuC), and ATPase (FeuD) involved in iron transport. This operon has also been reported in the nitrogen-fixing bacterium *Gluconacetobacter diazotrophicus* [36].

Seven genes of the iron-regulated surface determinant (Isd) system were found in the genomes of *L*. *sphaericus* TL, namely: *isdA*, *isdC*, *isdE*, *isdF*, *isdG*, *srtA*, and *srtB*. This system extracts heme group from hemoproteins and transports it into the cytoplasm, where free iron is released [37]. The genomes of *L*. *sphaericus* TL are also equipped with the HssRS/HrtAB heme detoxification system, which is responsible for preventing heme toxic levels in the cytoplasm.

### The toxic lineage of *L*. *sphaericus* is likely able to degrade aromatic compounds

Some genes previously reported as being involved in aromatics biodegradation were found in the *L*. *sphaericus* TL genomes. Three genes encoding for enzymes of the meta cleavage pathway were identified: the catechol 2,3-dioxygenase gene (*catE*), 4-oxolocrotonate tautomerase gene (*praC*), and the acetaldehyde dehydrogenase gene (*adhE*). Other genes involved in the catechol metabolism were found as well, such as the muconate cicloisomerase (*catB*) and the succinyl-CoA transferase (*scoA-B*). Interestingly, two genes involved in the degradation of sulfonated and hydroxylated aromatics were identified: the genes encoding for the enzymes toluene sulfonate zinc-independent alcohol dehydrogenase and 4-hydrophenyl acetate 3-mono-oxygenase.

## Discussion

*L*. *sphaericus* TL is a promising agent for biocontrol and bioremediation. Because of this, a comprehensive understanding of its metabolism is required in order to guide implementation strategies and further studies. In this paper, we present a metabolic reconstruction of *L*. *sphaericus* TL along with analysis about those traits that could be used in environmental and biotechnological applications.

The analysis of the *L*. *sphaericus* TL genome confirmed the high degree of conservation and synteny among the toxic strains, perhaps due to a genome stabilization after the acquisition of toxin-encoding genes as hypothesized previously [6]. This conservation allowed us to conduct an overall study of the *L*. *sphaericus* TL metabolism, because all the traits herein studied are shared by all the toxic strains. The commonly used approaches to evaluate evolutionary relationships among bacteria, such as phylogenetic reconstructions based on 16S rDNA sequences and core-genome alignment, do not provide sufficient resolution for the studied strains [6]. We achieved a better resolution by constructing alignment-free phylogeny based on K-string fixed peptides predicted from whole genome sequences [15]. This approach has been successfully used to reconstruct the phylogeny of bacteria and archaea, providing results comparable to those obtained by 16S rDNA or multi-locus alignments [14].

We generated a manually curated pathways database based on multiple genome annotations and on the pathways available on the MetaCyc database [38]. At the date of this work, there was a metabolic reconstruction for *L*. *sphaericus* C3-41 available on BioCyc. However, it is a model generated automatically and thus, may contain errors. We conducted a gene by gene revision of the pathways with an implication for bioremediation or biocontrol and proposed some annotations that differ from those uploaded on the NCBI database. The NCBI Prokaryotic Genome Annotation Process uses BLAST against a set database of RefSeq entries to name the predicted proteins [13]. Hence, it can be biased by the current annotation, which contain several proteins catalogued as hypothetical regardless the presence of a well-known domain architecture. We renamed several of these hypothetical proteins (S1 Table) on basis of domain architecture and on the best hit with reviewed entries on UniProt. All the reviewed proteins were assigned into metabolic pathways resulting in a reconstruction with a better theoretical and experimental consistency.

Our elucidation of the central metabolism of *L*. *sphaericus* TL is supported by experimental data published in the scientific literature. Hu and coworkers [21] published an exhaustive review about the carbohydrate metabolism in the toxic strain C3-41. The frameshifts or absent genes responsible for the lack of sugar metabolism identified by Hu and colleagues are the same that we found for the rest of the TL. Although the carbohydrate pathways are disrupted in several reactions, the glucose isomerisation to fructose is the first truncated step in these strains. This reaction is essential since the cleavage of glucose is not feasible due to the lack of a carbonyl group beside the cleavage site [39]. In view of this, an optimal medium for this bacterium must include a usable carbon source (glycerol or acetate), minimal required minerals, and at minimum, the amino acids mentioned above.

The obtained results suggest that carbon and nitrogen metabolism is nearly related to entomotoxicity. Since chitin is an essential structural component of the insect exoskeleton, the enzymes able to degrade this polymer are also important insecticidal factors [40]. Other bacteria such as *Bacillus thuringiensis* use this kind of enzymes, together with proteases and lipases, in a necrotrophic lifestyle to spread spores and complete their lifecycle [24]. Likewise, *L*. *sphaericus* TL may use chitinases to obtain acetate (carbon source) and N-acetylglucosamine (carbon and nitrogen source) from the insect tissues in a virulent lifestyle. Indeed, *L*. *sphaericus* TL has been the focus of many research projects precisely due to its enthomotoxic activity. This activity represents the great potential of *L*. *sphaericus* TL to be used as a biocontrol agent. In our search, we looked for genes involved not only in larvicidal activity but also in other kinds of entomotoxicity, such as those against nematodes, cockroaches and other plagues (Table 2).

Considering that all the reviewed insecticidal factors belong to different homology groups and thus are associated with different pathogenicity mechanisms and evolutionary origins [41], we claim that *L*. *sphaericus* TL can be considered a diverse reservoir of insecticidal genes. In addition to the well-known binary and mosquitocidal toxins, we reported the CryBP1 toxin, the sphaericolysin, and four hemolysins. These latter ones are able to lyse the insect immune cells granulocytes and plasmatocytes [22]. Moreover, some hemolysins are known to share structural homology to spore-forming toxins as Cry6A [42].

We proposed that an important step within the *L*. *sphaericus* TL lifecycle is a virulent stage inside the insect host. *L*. *sphaericus* TL would enter as spores and first attack epithelial cells using the binary toxin. After germination, it would use other toxins depending on the host (Mtx, Cry or Sphaericolysin) while suppressing the immune system using hemolysins to proliferate among epithelial cells, perhaps with a synergic activity of these factors. Additionally, bacterial cells would get nutrients by means of chitinolytic enzymes. The detailed regulatory mechanism for this activity is not fully understood and must be studied in detail.

Another important characteristic herein studied is the S-layer protein. It consists of a large glycoproteins assembly that covers the whole cell surface [29]. S-layer can carry out a dual role in entomotoxicity and metal resistance [25]. It is believed that bacilli contain several copies of S-layer encoding genes in their genome to perform recombinational regulation depending on the environmental stress. The recombination events would change the crystal symmetry of the protein, which would be directly related to its main function [29]. Our evidence bolsters this hypothesis because the *L*. *sphaericus* TL genomes show only one functional *slpC* gene but multiple *slpC* copies with deletions and frameshifts. To date, there is only one report of the S-layer protein symmetry in *L*. *sphaericus* TL (strain 2362) and no analyses that evaluate its relationship to environmental stress [29].

With regard to resistance to toxic metals, we found numerous experimental reports supporting the resistance of *L*. *sphaericus* TL towards Cd, Cr, As, and Pb [3,4]. Unfortunately, genomic evidence that bolsters these findings is scarce. Peña-Montenegro and colleagues [43] pointed out the putative resistance of the toxic strain CBAM5 against Ni, As, Cu, Mn, Te, Cd, and Zn. In the current study, we confirmed that these findings apply to all TL, and results also suggest a possible resistance to Al and the existence of operons for Fe, Mo, and Co metabolism. Separately, S-layer protein could provide *L*. *sphaericus* TL with the capacity to accumulate other metal cations, such as Au [44]. The adsorptive capability of S-layer protein is not metal-specific and thus, it is best determined through experimental assessments [29].

It is worth noting that the resistance against Al is proposed based on a putative aluminum resistance protein. Likewise, we identified several genes for metals and antimicrobials resistance as well as nitrogenase-related genes with no relation to other genes or regulons. These elements could be regulated by trans interactions or, on the contrary, correspond to non-functional genes due to evolutive or recombination events. Another hypothesis, that should be evaluated about these genes, is that they belong to a complex metabolic network that would comprise a whole microbial community. This is especially feasible for those genes related to factors that can be important in symbiotic interactions, such as nitrogenases and siderophores biosynthesis.

Importantly, our metabolic reconstruction also allowed us to infer some traits of *L*. *sphaericus* TL in regard with microbial interactions. Some Gram positive bacilli, especially those isolated from environmental samples, have been studied for their potential applications as biocontrol agents against Gram negative pathogens [45]. This promising feature is related to quorum quenching activity, which presumably has been developed by these bacteria as an evolutionary strategy against Gram-negative competitors in soil [46]. In short, quorum quenching is defined as the disruption of molecular communication among bacteria mainly by enzymes that hydrolyze the signal molecules [45]. This seems to be the case of *L*. *sphaericus* TL that, with three lactonases, could be used as biocontrol agent not only for insects, but for bacterial phytopathogens as well. Until now, only one of these lactonases has been experimentally evaluated [30].

Furthermore, the use of AI-2 would allow *L*. *sphaericus* TL to sense its environment and to modulate its gene expression depending upon the adjacent organisms. AI-2 is an interspecific signal molecule used by Gram positive and Gram negative bacteria [31]. In contrast, we did not find any gene involved in intraspecific quorum sensing. Gram positive bacteria usually synthesize oligopeptides as signal molecules [31]. The quorum sensing-regulated behaviors in *L*. *sphaericus* TL have been not determined yet but, taking into account evidence reported for other bacilli, could encompass virulence, oxidative stress response, motility, and cellular differentiation [47].

Motility is another important trait that we analyzed in the current study. *L*. *sphaericus* TL has all the required genes for flagellar assembly as well as several genes related to chemotaxis. Notably, we found a gene that encodes a methyl-accepting chemotaxis protein, whose importance has been previously demonstrated in the chemotactic response towards amino acids [32]. In spite of the presence of these motility genes, along with some antimicrobial resistance and hemolysin genes, we found neither genomic nor reported experimental evidence to suggest that *L*. *sphaericus* TL could be a potential human, animal or plant pathogen. These traits are related to insecticidal activity or mechanisms commonly found in soil microorganisms.

We evaluated the iron metabolism in *L*. *sphaericus* TL. Despite the essentiality of iron for nearly all forms of life, its availability in natural environments is limited due to the rapid aerobic oxidation of the ferrous ion and the insolubility of the ferric ion. Bacteria acquire iron by secreting siderophores [48]. Following iron capture, ferric siderophores are imported into the cell through siderophore-specific receptors anchored to the cell membrane in Gram positive bacteria [49] and then, transported by ATP-dependent transporters to the cytoplasm [50].

A putative homologue of the anthrachelin or petrobactin biosynthesis operon in *B*. *anthracis* was identified in the genomes of *L*. *sphaericus* TL. Petrobactin is a bis-catecholate, α-hydroxy acid siderophore, which constitutes one of the main siderophore-mediated iron transport systems in *B*. *anthracis* [51].

Several siderophore-specific receptors and ATP-dependent transporters were found in the genomes of *L*. *sphaericus* TL, especially transporters from the ATP-binding cassette (ABC), which mediate the import of essential nutrients and the export of toxic molecules [52]. The structural variability of siderophores requires several Fe-siderophore transport systems [35]. Thus, although this bacterium seems to produce only petrobactin, there are proteins that recognize at least other three exogenous siderophores: ferrichrome, enterobactin, and bacillibactin. This would allow *L*. *sphaericus* TL to take advantage of siderophores produced by other bacteria for iron incorporation.

Iron reacts with peroxides through the Fenton reaction to generate hydroxyl radicals, which are highly reactive and damaging molecules [53]. Hence, a tight regulation process is needed to prevent iron to reach toxic levels. The transcriptional repressor Fur is a common regulator of iron uptake and storage in many bacteria [34]. When the intracellular concentration of the ferrous ion is higher than some threshold level, Fur binds target genes on Fur boxes, repressing transcription. Nine Fur-binding boxes were found upstream of the start of ORFs encoding for iron transporters and some proteins involved in oxidative stress (Table 3). Thus, it is possible to hypothesize that the expression of those genes depends upon the ferrous ion availability in the cytoplasm since they seem to be Fur-regulated. It is worth noting that genes positively regulated by Fur do not usually have binding boxes in their 5´ regions [54], so the full repertoire of Fur-regulated genes in *L*. *sphaericus* TL remains to be elucidated.

Another putative mechanism for the control of iron levels in the cell was identified in the *L*. *sphaericus* TL genomes: The HssRS/HrtAB heme detoxification system. This system, which is shared by many members within the phylum Firmicutes, senses heme through HssRS and then pumps heme excess through HrtAB transporter [55,56]. It was previously hypothesized that the HssRS/HrtAB system is exclusive of pathogenic bacteria that infect hemoglobin rich vertebrate hosts. However, Schmidt et al. recently demonstrated that HssRS/HrtAB system functionally occurs in the insect pathogen *B*. *thuringiensis* [57]. Although insects do not usually use hemoglobin and hemoglobin-like molecules as oxygen-carrying proteins, cytochromes, which depends on heme as cofactor, are ubiquitous among insects [58]. Moreover, as *L*. *sphaericus* TL is pathogenic for some hematophagous insects, it is possible for it to encounters exogenous heme in the midgut of the insect host, for which the HssRS/HrtAB system could play a key role preventing heme-mediated toxicity.

Besides, the Isd system was first described in *Staphylococcus aureus*, encompassing nine proteins (IsdA-IsdI) responsible for heme group extraction from hemoproteins and further incorporation of heme-iron into the cytoplasm. IsdA, IsdB, and IsdH are usually anchored to cell wall peptidoglycan by sortase A, whereas IsdC is anchored by a type B sortase [59]. These proteins contain one or more near iron transport (NEAT) motifs, which bind heme groups.

The *isdA*, *isdC*, *srtB*, and *srtA* genes were found in the genomes of *L*. *sphaericus* TL but there was no evidence for *isdH*, *isdD*, and *isdB*; the latter two have been observed only in the *S*. *aureus* genome. Hence, IsdA and IsdC seem to play a key role in providing iron in the intracellular space by binding heme and then passing it to the IsdEF complex, which pumps the heme group across the membrane [60,61]. In the cytoplasm, the heme oxygenases (IsdG and IsdI) break the porphyrin ring, releasing free iron [62] (Fig 3).

Lastly, we searched for genes involved in aromatics degradation. Some genes from the meta cleavage pathway of catechol were identified, including the gene encoding for the catechol 2,3-dioxygenase, the enzyme responsible for the cleavage of the catecholic ring. The meta cleavage pathway is a common catabolic process in aerobic organisms for the consumption of aromatic compounds. In the classical aerobic catabolism, the aromatic ring is activated through hydroxylation or oxygenation to catecholic intermediates that are further cleaved and converted to metabolites that can easily enter in the tricarboxylic acid [63]. In spite of the growing amount of reports of *L*. *sphaericus* strains able to degrade aromatics and other xenobiotics [64–67], there are no detailed studies that aim to elucidate the molecular mechanisms underlying this functional feature.

As concluding remark, a metabolic reconstruction based on gene annotation could be considered as an important contribution to the understanding of an organism. However, it is only an initial step since other factors must be considered. Usually, the genes involved in metabolic pathways do not exceed 30% of the total gene content [8]. Then, studies aimed to elucidate regulatory mechanisms and to characterize the *L*. *sphaericus* TL transcriptome are still necessary in order to achieve a comprehensive view of this bacterium. Likewise, further studies should confirm the putative functions herein attributed to some genes: The implication of CryBP1, sphaericolysin, and the hemolysins in *L*. *sphaericus* TL as biocontrol agent is not understood and, indeed, its lifecycle must be elucidated. The resistance to aluminum, lead, and chromium could not be confirmed through bioinformatics. Only one of the lactonases herein identified has been characterized, the other two lactonases should be studied in order to assess the potential of *L*. *sphaericus* TL as biocontrol agent for bacterial pathogens. The restriction-modification systems also should be examined. The methylation motifs files are available on the NCBI database for the four *L*. *sphaericus* TL genomes sequenced by PacBio. Similarly, predictions of those systems are available for six *L*. *sphaericus* TL strains on REBASE [68].

Finally, the available genomes of *L*. *sphaericus* TL offer an accurate representation of this conserved group of bacterial strains. Our metabolic reconstruction and annotations are consistent with the experimental evidence reported previously. Taking these results together, we claim that *L*. *sphaericus* TL is a potential biocontrol and bioremediation agent, and strategies for its exploitation should be designed and implemented. We also proposed new potential uses of *L*. *sphaericus* TL that must be tested experimentally.

## Methods

### Gene content analysis

This analysis was conducted with the 6 *L*. *sphaericus* TL genomes listed in Table 1. We used the currently available annotation on the NCBI Genome database and generated new annotations using Prokka [12] and the functional annotators RAST [17,18] and KAAS [19].

Prokka was implemented with the default parameters adding the “Prokka Kingdom Bacteria” database into the subject database set. The annotation by RAST catalogues the genes into subsystems according to functional categories. We performed this annotation with the default parameters. For KAAS, we used the Complete Genome function, which uses the bidirectional best hit method by BLAST (against the manually curated KEGG GENES database) to assign orthologs into KEGG Orthology groups and KEGG pathways. Prokaryotes was fixed as representative set adding “lsp” (for *L*. *sphaericus* C3-41).

### Phylogenetic inferences

The phylogenetic analysis was conducted by a free-alignment approach using whole genome sequences from the highly toxic strains and the strains OT4b.31 (WGS AQPX01), KCTC 3346 (WGS AUOZ01), NRS 1693 (WGS JPDM01), B1-CDA (LJYY01), 1987 (WGS JMMU01), SSII-1 (WGS JPDK01), 2297 (WGS JPDJ01), and *Solibacillus silvestris* StLB046 (AN AP012157) as outgroup. This approach was carried out using the CVTree 3.0 online server [14]. We constructed phylogenetic trees for 6, 7, 8, and 9 peptides strings using the FAA output (predicted proteins) generated by Prokka. The trees were consistent to each other.

Summarizing, this method is a systematic way to infer evolutionary relatedness among microorganisms based on the frequency of a fixed length (from 1 to 9 aa) oligopeptides in their complete proteomes. This approach circumvents the ambiguity of choosing genetic markers and avoids the necessity of aligning sequences with different length and gene content [15].

### Metabolic reconstruction

Pathway Tools and Pathologic software (version 20.0) [20] were used to automatically generate a pathway-genome database (PGDB) from the complete genome of *L*. *sphaericus* 2362 using the MetaCyc database as reference [38]. The Pathologic software makes an association between genes and reactions by means of EC numbers and function descriptions/names in the GenBank file of the annotated genome. The obtained PGDB consists of the complete genome content of *L*. *sphaericus* 2362 and connections between CDS, potential genes, pseudogenes, enzymes, reactions, and biochemical pathways.

Afterwards, the PDGB was manually polished for the pathways analyzed in this work by assigning probable enzymes, rescoring pathways, creating protein complexes, assigning modified proteins, parsing transport identification, filling pathway holes, and predicting transcription units. These processes are incorporated as options in the Pathologic software. The decisions made in these processes were supported by the results of functional annotation described below.

### Functional annotation

The annotations obtained from NCBI, Prokka, RAST, and KAAS were contrasted for those genes involved (even in only one annotation) in the studied pathways. If the annotation was consistent in all the annotations, this remains unchanged in the reported annotation (S1 Table). For some genes, discrepancies between two or more annotations were found. In such a case, the domains and the domain architecture of the encoded protein sequence were analyzed using the CDD/SPARCLE function classification [69] and Pfam [70]. Moreover, the sequence was blasted against the UniProt database looking for the best manually reviewed hit with a significant e-value (< 1e-6). When the discrepancy was “hypothetical protein / uncharacterized protein / unnamed protein” vs. a well-defined annotation, the latter annotation was assigned because in all the reviewed cases, it was supported by the analyses described above. When the discrepancy was a general annotation vs. a specific annotation (e.g. Transporter vs Iron Transporter), in addition to the previous analyses, the probability of belonging to a known regulon (according to adjacent genes) was evaluated using the RegPrecise database as an approach to the genetic context [28].

### Search for potential Fur binding sites

Putative Fur binding sites within the genome of *L*. *sphaericus* TL were determined using a BLAST-n search automatically optimized for short sequences. The query sequence was the conserved Fur binding site in *B*. *subtilis*: TGATAATNATTATCA as previously determined by Fuangthong et al. [33]. We only accepted hits that were in intergenic spaces within 250 pb of an identified ORF and contained a maximum of two mismatches from the consensus sequence.

### Phenotypic evaluation

A comparison between inferred pathways and experimental evidence was carried out by bibliographic review. Lipolytic and proteolytic activity of the toxic strains 2362, OT4b.25, OT4b.49, and CBAM5 was assessed as described previously [24]. These strains are routinely cultured in Luria Bertani medium at 30°C.

The minimum inhibitory concentrations (MICs) of erythromycin, tetracycline, trimethoprim, gentamicin, kanamycin, rifampicin, ampicillin, and chloramphenicol were determined by broth dilutions in BHI. This experiment was conducted three times for the strains listed above, with a standardized inoculum of 10^5^ UFC/mL and incubating at 30° C for 24 h. As the results were the same for all the strains, the MICs values were collapsed in S2 Table.

### Illustrations

The output from RAST (Fig 2) was illustrated using the plotly package in the R Statistical Software [71]. The phylogenetic tree from CVTree 3.0 was illustrated using MEGA 6.0 [72]. The metabolic overview representation (Fig 3) was constructed based on the manually curated annotation (S1 Table) using ChemBioDraw® Ultra 12.0 Suite by Cambridge Soft.

## Supporting information

S1 Table Reviewed gene annotation of *L. sphaericus* TL. This table contains the genes reviewed in this work, which are related to the studied metabolic pathways and traits. The genes are ordered and depicted in a certain color according to their relation to a metabolic pathway. We indicated the RefSeq entry for each predicted protein as it is the same for all the toxic strains. Asterisks (*) in the observations column indicate a discrepancy between the proposed and NCBI annotations. Whether the genes are arranged as an operon is also indicated in the observations column.
(XLSX)

S2 Table *L. sphaericus* TL antibiotic susceptibilities. (XLSX)
